# Toward new health and welfare policies to overcome low birth in Korea

**DOI:** 10.4069/kjwhn.2022.03.16

**Published:** 2022-03-30

**Authors:** Sukhee Ahn

**Affiliations:** College of Nursing, Chungnam National University, Daejeon, Korea

## Introduction

In the population policy of South Korea (hereafter, Korea), suppression of population growth was first introduced in 1961, in line with the First Five-Year Economic Development Plan (1962–1966). However, in response to the opinion that continuous implementation of this suppression policy would not be conducive to socioeconomic development in the long term, it was abolished in 1996 and the direction of the population policy was changed to a focus on population quality and welfare policy [1]. After setting low fertility and aging society on the national agenda in 2003, Basic Plans were established and promoted over the years: the First Basic Plan on Aging Society and Population (2006–2010), followed by the Second (2011–2015) and the Third (2016–2020) Basic Plans [1,2]. Despite such an explosion of policies to encourage childbirth over the past 15 years, however, the outcome was below expectations. Because only a few households were willing to give birth with financial support, the effectiveness of the policy was only temporary, and there was a limit to a sustainable increase in the birth rate over the long term. Similar patterns have occurred in East Asian countries such as Japan, Taiwan, and Hong Kong, in terms of low birth rate and limited impact of policies aimed to increase it [3].

Concerns over population decline are growing serious in Korea, as the total fertility rate—the average number of children that would be born to each woman over her lifetime—stood at an all-time low of 0.81 in 2021, a further drop from 0.84 in 2020 [4]. In response to these concerns, the paradigm of the low fertility policy has shifted from encouraging fertility *per se* to improving quality of life, i.e., focusing on the social structural ‘causes’ that led to low fertility and seeking to resolve them to improve overall quality of life [4]. This paper will review causes of low birth rate and new policies toward “improving quality of life” outlined in the Fourth Basic Plan (2021–2025), and make suggestions for the successful implementation of the policy.

## Causes of low birth rate in Korea

Korea’s total fertility rate marked the fourth straight year (2018–2021) of a rate below 1%. Furthermore, in 2019 the first natural decline in population was reported, as the number of deaths outpaced that of newborns [5], and subsequently concerns over population policy are deepening. Several factors have been posited to explain the persistence of low birth rate in Korea. Socioeconomic factors include large gaps and gender discrimination within the labor market, increasing competition in education, expensive housing prices, and burden to child-rearing families due to lack of child care support. It is difficult for young people to find a stable and secure job in terms of full-time work with adequate salary. Korea’s labor market structure with gender discrimination exists in terms of employment rate, wage level, and job quality is also one of the causes. This precarious labor market causes intensifying competition in education. In addition, the burdens of housing and education expenses, lack of child care, and a system that is not employee-friendly become obstacles for married dual-income couples to consider having children [2,4-6].

People’s values toward marriage and children has changed over the years and life priorities have shifted from marriage and children to work in Korea’s younger generation. A recent survey of young Korean adults in their 20s to 30s [7] found that their life perspective is becoming de-gendered, i.e., both men and women reported the importance of their life tasks in the following order: work taking highest priority, followed by personal life, partnership, and children. Women are designing their life course centered on a ‘work-centered life’ rather than a ‘family-centered life’, although there was no significant difference between young women and men of the same age. This survey also reported that young Korean men feel pressured with work and precarious living conditions, as much as women do; nevertheless, men were also supportive of the prospect of participating in nurturing.

Ultra-low fertility is a serious issue, not only in Korea, but also in several East Asian countries, which has been attributed to gender inequality [3]. Difficulty reconciling work and family life in Korean society are major obstacles and career interruption due to childbirth and child-rearing prevents both women and men from having a stable working life. The difficulty in balancing childbirth/parenting and maintaining a dual-income life, subsequently presents as both men and women tending to avoid marriage and childbirth. When examining a list of what young Koreans perceive as prerequisites for having children, women showed the highest degree of agreement with ‘partner’s participation in child-rearing,’ ‘fair household burden,’ and ‘spouse’s maternity and parental leave,’ which ranked higher than economic stability or the woman’s own sense of work-family balance. It shows a big difference from men who mainly responded based on economic requirements [7].

The economic crisis and the coronavirus disease 2019 (COVID-19) pandemic have also impacted a further decrease in birth rate. For example, there are serious problems of inequality in employment, increasing demands for expanding social safety nets, and a vacancy in childcare options due to temporary suspension of childcare centers and schools because of concerns about the spread of COVID-19 infection [8].

## New policies toward “improving the quality of life” with the Fourth Basic Plan (2021–2025)

The social shifts described above strongly suggest that rather than focusing on birth rate *per se*, the focus should be on improving the quality of life of the younger generation in their 20s to 40s, who are planning marriage and considering having and raising children. The Korean government announced the Fourth Basic Plan (2021–2025) in December, 2020 [4]. The vision of this plan is creating a “sustainable society where all generations are happy together” with three main goals: improving individual quality of life, establishing a gender-equitable and fair society, and social innovation in response to demographic changes. Four strategies are outlined as follows: (1) creating a society where we work together and care for each other, (2) building a healthy and active aging society, (3) establishing a society where everyone’s capabilities are recognized, and (4) adapting to leap forward in response to demographic changes [6].

To this aim, the Korean government launched the ‘Five major packages for overcoming the low birth rate’ to ease the burden of pregnancy, childbirth, and child care [4]. The five packages include providing infant allowance, a ‘first meeting’ package, expansion of multi-child support, activation of parental support for couples, and expansion of public childcare centers. The first package offers intensive support from pregnancy to early childhood, with government subsidies of 300,000 Korean won (KRW; approximately 240 US dollars) per month for infants from birth to 1 year of age starting in 2022. This infant allowance will be raised in stages up to 500,000 KRW (approximately 400 US dollars) by 2025. Second, a ‘first meeting package’ worth 3 million KRW (approximately 2,700 US dollars) is given to families who have given birth. Third, multi-child support is extended from ‘third child or more’ to the second child and discounts are available on childcare, education, housing and travel costs. The fourth package seeks to establish a comprehensive and high-quality care system people can trust in with confidence, such as expanding public childcare and after-school day care. The final package aims at strengthening work-life balance, such as increasing payment levels for parental leave and flexibility in working hours, and more investment in the well-being of families. This focuses on strengthening systems not only for financial support and policy, but for childcare services as well. In addition, the basic plan includes the task of ‘institutional acceptance of diverse families’ into the ‘adaptation to demographic change’ area. As such, respect for family diversity is presented as a major future-oriented task.

## Suggestions for successful policy implementation

The declaration that the Fourth Basic Plan will move from a “national-centered perspective” toward “individual quality of life” is judged to be a very important and meaningful policy paradigm shift [2,6,9]. Nevertheless, there may be limitations in responding to the issue of low birth rate itself, as most support policies are focused on married families planning to have or raising children. There is a need for policy to pro-actively aim at resolving imbalances in the labor market by expanding the supply of high-quality jobs for the young unmarried generation to have stable employment; and reducing distortions in the cost structure that has heavily focused on housing and education [10]. Policies should include strategies for accommodating economic and social environmental changes and adapting to the expected future demographic changes.

As an explanation for the second-half of the gender revolution, Goldscheider et al. [11] proposed that men’s participation in caring roles and a greater change in the perception of gender equality would lead to an equal partnership and a change in the fertility rate. Given that both Korean young men and women overwhelmingly support the gender equality model regarding the division of labor between work and family [7], the Korean government should support a gendered perspective. Further, young Korean women are more sensitively aware of the need for family-inclusive systems and reproductive rights [7]. This is similar to the report that the social structure and institutions in all dimensions from work to life should be rebalanced toward attaining higher quality of family life, which is hoped to lead to higher motivation to have children [10]. As such, new policy approaches need to incorporate these life perspectives for pursuing a gender-equitable society and improving quality of life for everyone.

A large proportion of young Korean women surveyed agreed with the need for safe contraception and abortion support [7]. The Fourth Basic Plan includes sexual and reproductive rights and set three subtasks with comprehensive guarantee of sexual and reproductive rights, lifelong reproductive health management and disease prevention, and guarantee of healthy and safe pregnancy and childbirth [6]. Although it appears to be freeing itself from excessive focus on the ‘birthing body,’ the plan did not include women’s overall reproductive health problems nor the right not to have children [6,9,12]. Given these limitations, policies on expanding support for women’s bodies need to be added in the future.

While housing is one of the barriers to marriage and childbirth for the young generation and newly married couples, strategies to address this are still limited [6,13]. Therefore, future efforts should establish a policy for housing cost assistance, such as public rental housing that meets the needs of young people, through cooperation among various government ministries.

In conclusion, it is necessary to shift the focus from the national point of view that low birth is a problem, to the individual point of view that values and enhances quality of life, so that the prospect of having and raising children can be naturally supported. Ongoing challenges for the Korean government include designing and implementing effective, efficient, integrated, and long-term policies based on needs analysis. The new government, which will be launched in May 2022, following the 15th Presidential Election, pledged to abolish the Ministry of Gender Equality and Family, amid many concerns [14]. However, the new government-elect should make efforts through inter-ministerial policy coordination to ensure that diverse population policies for gender equality continue without setbacks.

To solve the problem of low birth rate in Korean society, (1) the social perception that women bear main responsibility for childbirth and child rearing needs to be changed, (2) the burden of childcare should not be shifted solely to the individual/family, and (3) low fertility should not be approached as an individual problem, but a social problem that the nation must respond to. When individuals, families, workplaces, and communities all develop into a gender-equitable society that fully supports work-family balance, members of society will be able to choose marriage, childbirth, and child rearing while pursuing a high quality of life. Nurses should highlight their competence as healthcare professionals by actively participating in the latest policy proposals and projects to overcome the low birth rate. In addition, nurses can actively participate in the establishment of policies on women’s reproductive rights and raise their voices as advocates for women’s right to health.

## References

[1] http://repository.kihasa.re.kr/handle/201002/36278.

[2] Woo HB (2020). Future demographic change and policy direction. Health Welf Policy Forum.

[3] Jones GW (2019). Ultra-low fertility in East Asia: policy responses and challenges. Asian Population Studies.

[4] https://www.betterfuture.go.kr/front/policySpace/basicPlanDetail.do?articleId=105.

[5] https://www.mk.co.kr/news/economy/view/2020/01/96194/.

[6] Kim EJ (2021). The fourth basic plan for low fertility and aging society. Gender perspective. Issue Brief.

[7] Kim EJ, Song HR, Bae HJ, Sun BY, Choi JH, Hwang JM (2019). Paradigm shift in policy responses to low birthrates (Ⅰ): an analysis of the gendered life perspective and policy validation of youth.

[8] Kim GB (2020). Turn of civilization in the post COVID-19 era and population policy. Hist-Cult Res.

[9] Cho KA (2021). Korea’s low birth rate issue and policy direction. Korean J Women Health Nurs.

[10] Kang DS (2020). Report of integrated policy in response to the low birth rate.

[11] Goldscheider F, Bernhardt E, Lappegård T (2015). The gender revolution: a framework for understanding changing family and demographic behavior. Popul Dev Rev.

[12] Noh JH (2021). The evolvement of sexual and reproductive health policies in Korea. Korean J Women Health Nurs.

[13] https://www.nabo.go.kr/Sub/01Report/01_01_Board.jsp?bid=19&item_id=7569&arg_id=7569&funcSUB=view.

[14] https://www.npr.org/2022/03/08/1085099933/gender-equality-has-become-a-polarizing-issue-in-south-koreas-presidential-elect.

